# Saliva exosomes-derived UBE2O mRNA promotes angiogenesis in cutaneous wounds by targeting SMAD6

**DOI:** 10.1186/s12951-020-00624-3

**Published:** 2020-05-06

**Authors:** Bobin Mi, Lang Chen, Yuan Xiong, Chenchen Yan, Hang Xue, Adriana C. Panayi, Jing Liu, Liangcong Hu, Yiqiang Hu, Faqi Cao, Yun Sun, Wu Zhou, Guohui Liu

**Affiliations:** 1 Department of Orthopedics, Union Hospital, Tongji Medical College, Huazhong University of Science and Technology, Wuhan, 430022 China; 2 Department of Plastic Surgery, Brigham and Women’s Hospital, Harvard Medical School, Boston, MA USA; 3 Department of Neurosurgery, Union Hospital, Tongji Medical College, Huazhong University of Science and Technology, Wuhan, 430022 China

**Keywords:** Saliva, Exosome, UBE2O, Wound, Angiogenesis, SMAD6

## Abstract

**Background:**

Enhancing angiogenesis is critical for accelerating wound healing. Application of different types of exosomes (Exos) to promote angiogenesis represents a novel strategy for enhanced wound repair. Saliva is known to accelerate wound healing, but the underlying mechanisms remain unclear.

**Results:**

Our results have demonstrated that saliva-derived exosomes (saliva-Exos) induce human umbilical vein endothelial cells (HUVEC) proliferation, migration, and angiogenesis in vitro, and promote cutaneous wound healing in vivo. Further experiments documented that Ubiquitin-conjugating enzyme E2O (UBE2O) is one of the main mRNAs of saliva-Exos, and activation of UBE2O has effects similar to those of saliva-Exos, both in vitro and in vivo. Mechanistically, UBE2O decreases the level of SMAD family member 6 (SMAD6), thereby activating bone morphogenetic protein 2 (BMP2), which, in turn, induces angiogenesis.

**Conclusions:**

The present work suggests that administration of saliva-Exos and UBE2O represents a promising strategy for enhancing wound healing through promotion of angiogenesis.

## Background

Worldwide prevalence of chronic wounds is increasing each year due to aging of the population [1]. Chronic wounds not only impact the health and quality of life of patients but also pose a significant socioeconomic burden for the entire healthcare system [2]. Wound healing comprises of a well-orchestrated sequence of events, of which the process of angiogenesis is essential for nutrient and oxygen delivery to cells in the wound [3]. An increasing number of studies have provided strong evidence that enhanced angiogenesis can effectively accelerate wound healing [4, 5]. Thus, promotion of angiogenesis and neovascularization in the wound has become the focus of intense research.

Numerous proteins and growth factors contained in saliva make it a promising source of factors promoting tissue regeneration [6, 7]. However, the use of saliva as an effective agent to promote local health has not yet gained widespread acceptance. The unique active constituents derived from saliva have attracted attention in the field of biomaterials, and exosomes (Exos) present in the saliva appear to be of particular significance [8, 9]. Exos are small vesicles enriched with bioactive molecules such as lipids, proteins, mRNAs, and miRNAs [10]. Exos, containing these molecules, can be transferred into target cells to affect cellular functions [11, 12]. Given the multiple effects of Exos on cell activity, it would be interesting to investigate whether saliva-derived exosomes (saliva-Exos) can positively effect cells participating in wound repair, accelerating this process in vivo.

Based on this, the objective of the present study was to explore the role of saliva-Exos on the function of vascular endothelial cells and to uncover the underlying mechanisms. Additionally, experiments were performed to determine the effects of saliva-Exos on cutaneous wound healing in vivo.

## Results

### Identification of saliva-Exos

**Fig. 1:**
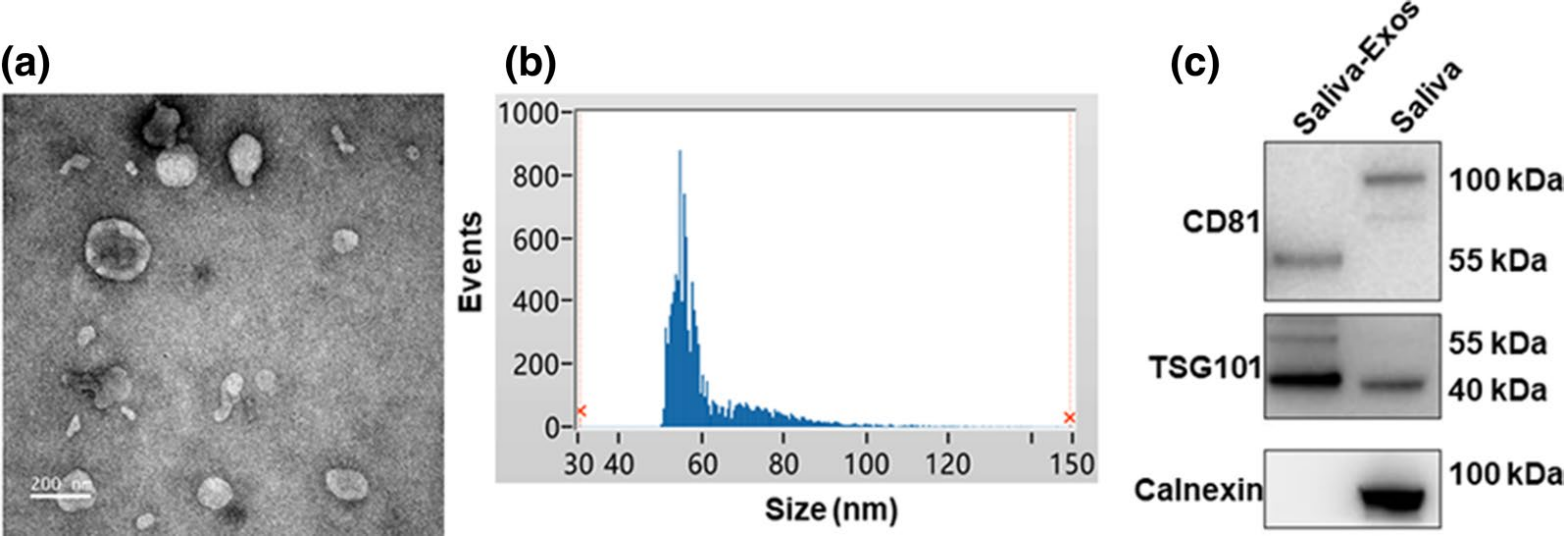
Characterization of Saliva-Exos. **a** TEM image of saliva-Exos. **b** Size of saliva-Exos measured using NanoSight. **c** Western blot results showed that the enriched TSG101 and CD81 level and lower TSG101 level in the saliva-Exos sample compared to the saliva. Exosome-specific markers TSG101, CD81 and non-exosomal marker calnexin detected by Western blotting

The saliva-Exos were identified and characterized by transmission electron microscope (TEM), NanoSight analysis, and Western blotting. In agreement with previously reported results for Exos, the TEM and NanoSight analysis demonstrated that the size of the isolated particles ranged from 30 to 150 nm (Fig. 1a, b). Western blotting documented that the particles contained enriched proteins CD81 and tumor susceptibility gene 101 protein (TSG101), but did not contain calnexin (Fig. 1c), confirming that the Exos were successfully isolated from the salivary samples.

### Saliva-Exos accelerate cutaneous wound healing in vivo

**Fig. 2:**
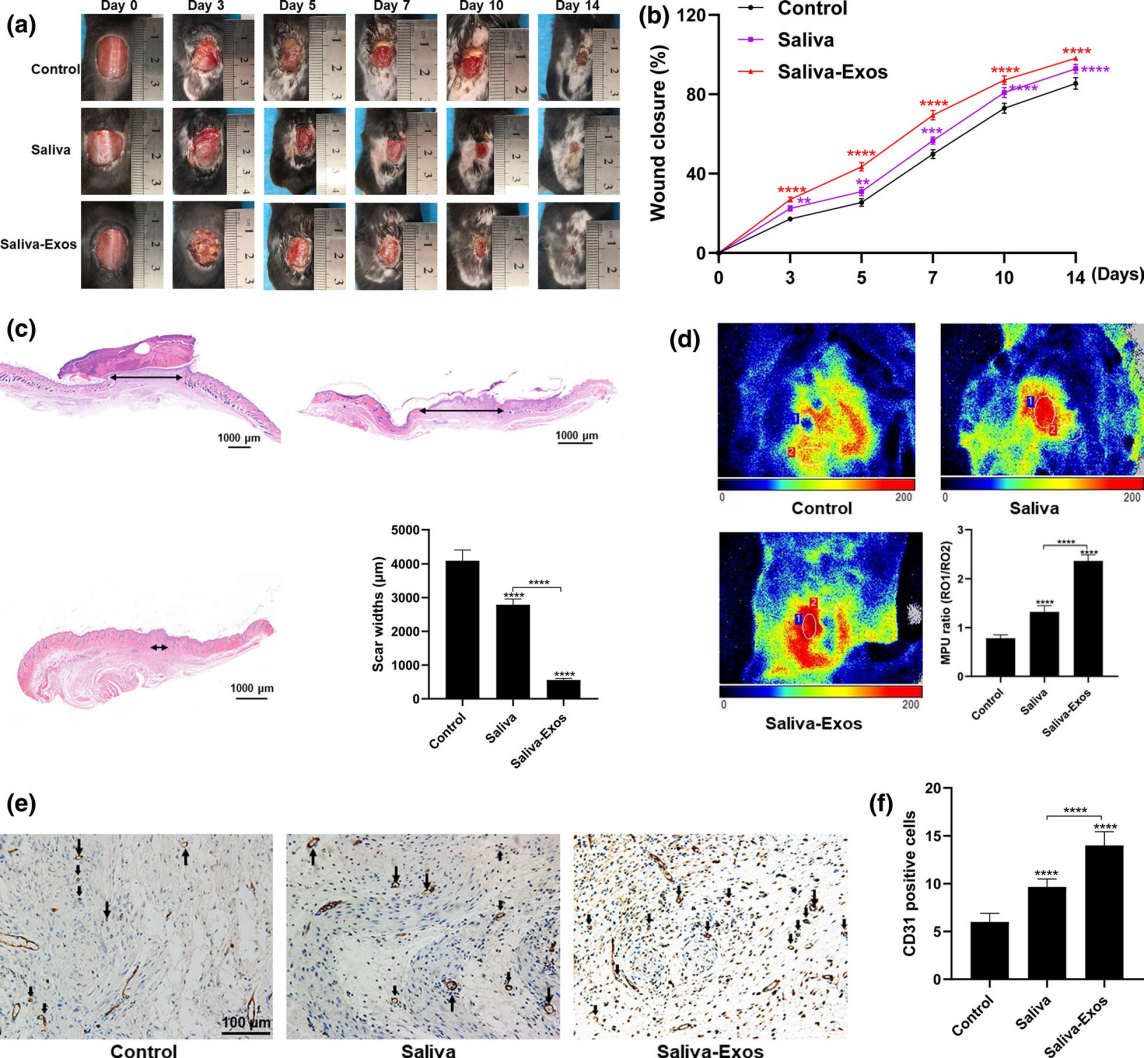
Saliva-Exos accelerate wound healing in vivo. **a** Images of cutaneous wound in mice. **b** Wound healing rate in the three in vivo groups, mice treated with PBS, saliva, and saliva-Exos. **c** H&E staining of wound sections and quantification of scar widths in the three groups. **d** Blood flow in the wound site assessed by laser speckle contrast imaging and quantification of MPU ratio in the three groups. **e** Representative images of CD31 staining of wound sections. **f** Quantification of the number of CD31-positive cells in the three groups. **p < 0.01, ***p < 0.001, ***p < 0.0001; n = 6 per group

To determine the role of saliva-Exos in wound repair, equal amounts of phosphate buffer saline (PBS), saliva, and saliva-Exos were injected around the wound site. The saliva and saliva-Exos groups had a higher rate of wound healing than the control group, with the process being faster in animals treated with saliva-Exos than saliva only (Fig. 2a, b). The scar width was smaller in the saliva-Exos group than in the control group (Fig. 2c). The neovascularization of the wound site was significantly higher in the saliva-Exos group, as documented by increased blood flow (Fig. 2d) and higher number of CD31-positive cells (Fig. 2e, f). Together, these findings indicate that saliva accelerated wound healing, and this effect can be attributed to the promotion of angiogenesis by saliva-Exos.

### Saliva-Exos enhanced the function of HUVECs

**Fig. 3:**
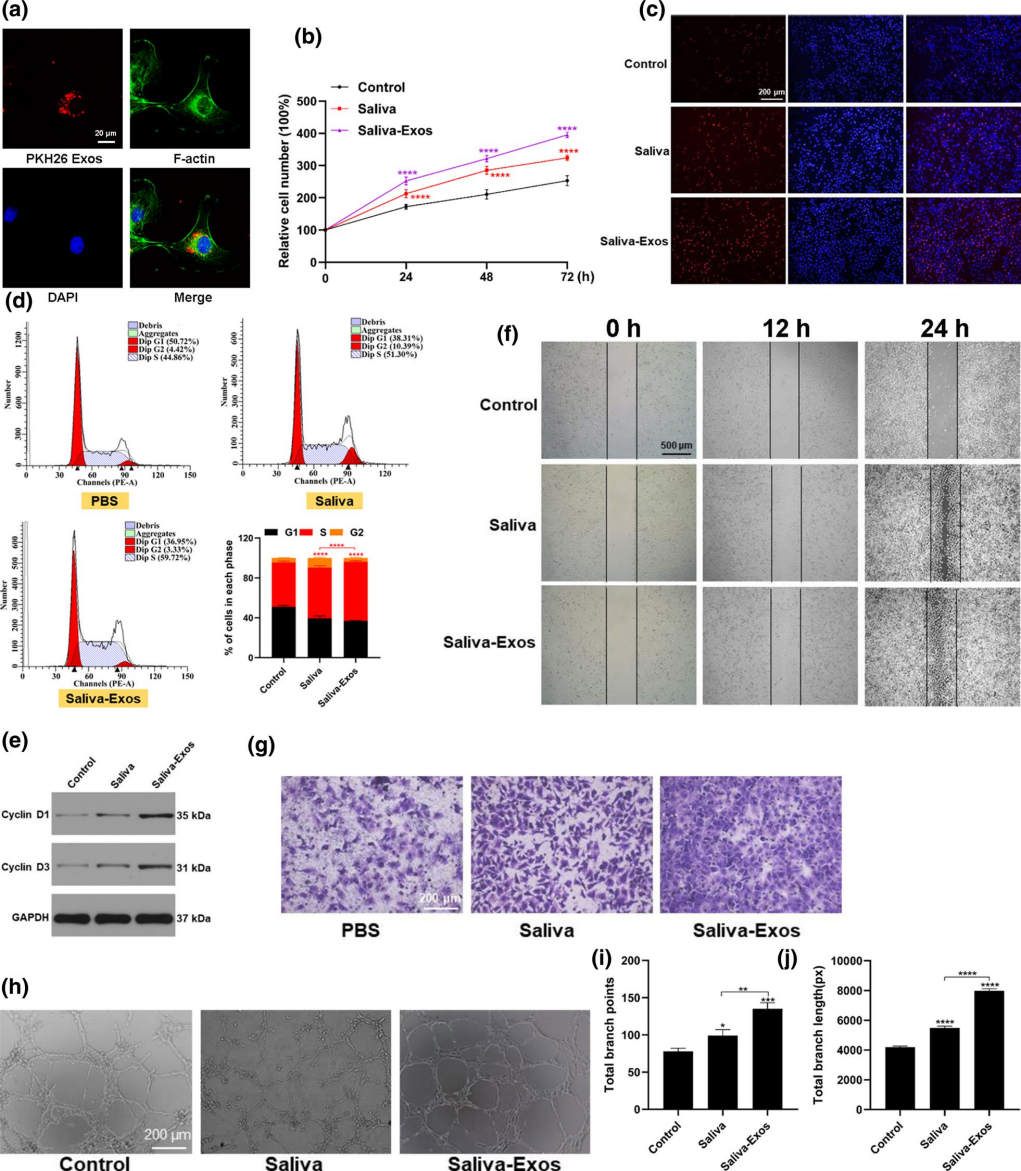
Saliva-Exos enhanced the function of HUVECs. **a** Saliva-Exos were labeled with PHK26 and added to a HUVEC culture. The presence of a fluorescent signal inside the cells documents that saliva-Exos were taken up by HUVECs. **b**, **c** CCK8 and EdU assay were performed after HUVECs were treated with PBS (control), saliva, or saliva-Exos. **d** The cell cycle was assessed by flow cytometry. **e** Western blotting was used to evaluate the expression of the cell cycle-related proteins, Cyclin D1 and Cyclin D3. **f**, **g** Wound scratch assay and transwell assay were used to evaluate migration of HUVECs after treatment with PBS (Control), saliva, or saliva-Exos. **h** Representative images of the tube formation assay in HUVECs treated with PBS, saliva, saliva-Exos. **i**, **j** Quantitative analyses of total branch length and total number of branching points in the three groups. *p < 0.05, **p < 0.01, ***p < 0.001, ****p < 0.0001

The effect of saliva-Exos on human umbilical vein endothelial cells (HUVECs) in vitro was assessed. To determine whether Exos can be taken up by HUVECs, saliva-Exos were labeled with the fluorescent dye PKH26 and added to the medium of HUVEC cultures. After 12 h of incubation, the Exos were successfully transferred into the cells (Fig. 3a). To determine the impact of saliva-Exos on HUVEC proliferation, the cells were treated with equal volume of PBS, saliva, or saliva-Exos. Cell count kit 8 (CCK-8) and EdU assays revealed that saliva-Exos promoted the proliferation of HUVECs (Fig. 3b, c). Saliva-Exos induced higher percentage of HUVECs to enter the S stage (Fig. 3d) and up-regulated the expression of cell cycle-related proteins (Fig. 3e). HUVECs treated with saliva-Exos exhibited higher rate of migration than cells in the control group (Fig. 3f, g). Additionally, saliva-Exos induced tube formation by HUVECs (Fig. 3h–j). Collectively, these data indicate that saliva-Exos play a positive role on HUVEC function.

### UBE2O is the hub gene on saliva-Exos upregulated genes

**Fig. 4:**
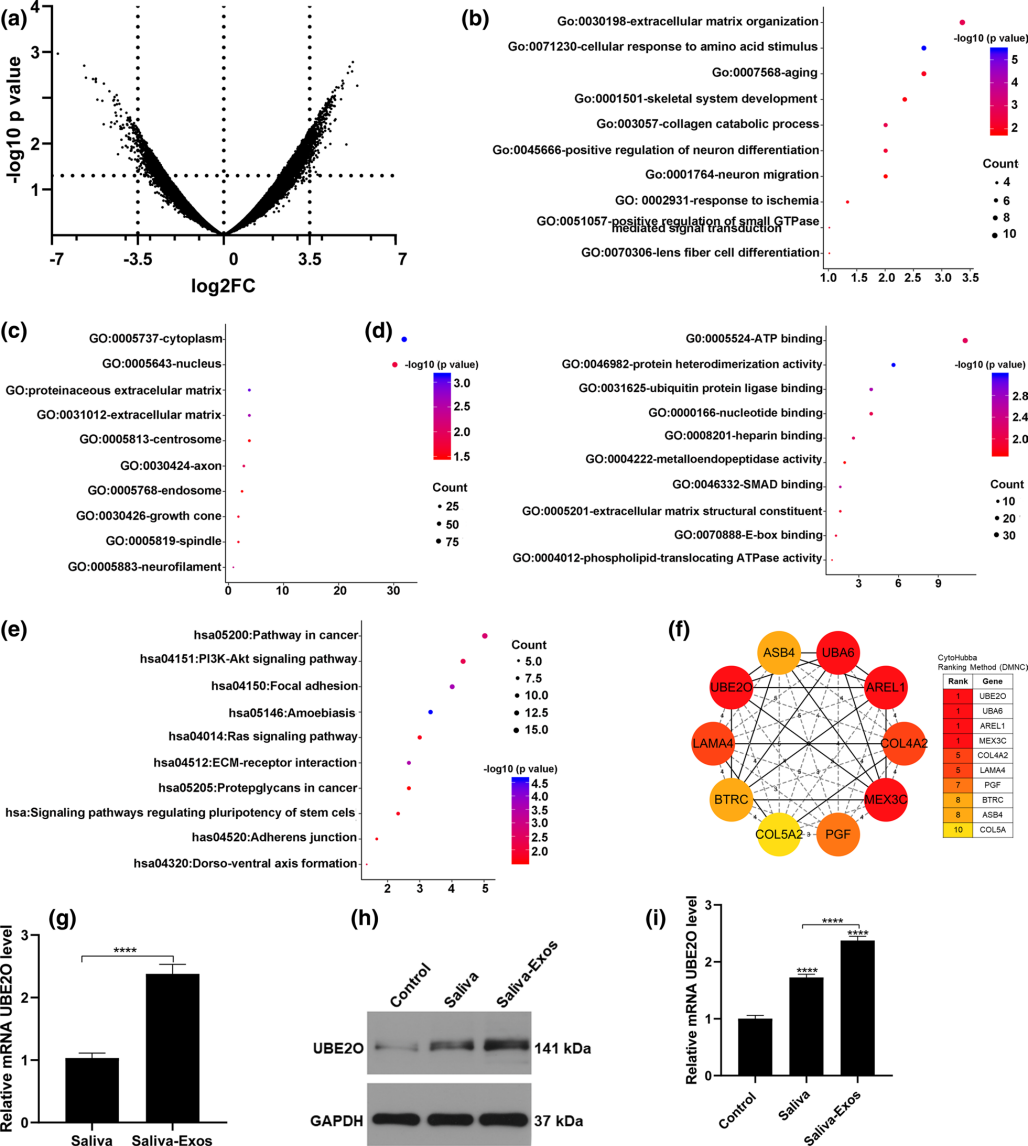
UBE2O is the hub gene on saliva-Exos upregulated genes. **a** Valcano plot illustrating the differential mRNA expression level between saliva and saliva-Exos. The negative Log10 p values (y axis) are plotted against the Log2 fold changes in expression (x axis). **b**–**e** Analysis of biological process (BP) (**b**), cellular Component (CC) (**c**), molecular function (MF) (**d**) and KEGG pathway (**e**) of upregulated genes, the bubble diagram generated using the ggplot2 tool of the R software showing the top ten pathways. (**f**) Identification of UBE2O as one of the main hub genes using the DMNC algorithm to screen the upregulated genes. (**g**) UBE2O mRNA level in saliva and saliva-Exos assessed by qRT-PCR. n = 5. **h**, **i** UBE2O expression in HUVECs treated with PBS (control), saliva, and saliva-Exos, detected by Western blotting and qRT-PCR. ***p < 0.001, ****p < 0.0001

To explore which molecular pathways mediate the beneficial effects of saliva-Exos on HUVECs, the RNA-sequencing data for saliva and saliva-Exos were extracted from the GEO database (GSE50700) and screened for the differentially expressed genes (DEGs) among the two groups (Fig. 4a). After applying the criteria of Log2FC ≥ 3.5 and P-value < 0.05, 312 upregulated DEGs in saliva-Exos were screened. The tissue-specific protein-protein interactions (PPI) network between the 312 upregulated DEGs with their putative targets in skin-tissues were constructed using the online tool NetworkAnalyst (https://www.networkanalyst.ca/) (Additional file 1: Fig. S1A). Subsequently, the Database for Annotation, Visualization and Integrated Discovery (DAVID) platform was used to analyze the functional-enrichment analyses including GO and Kyoto encyclopedia of genes and genomes (KEGG) pathway enrichment of these 312 genes. The top ten GO enrichments and KEGG pathways are shown in Fig. 4b–e.

To establish the hub genes, we used density of maximum neighborhood component (DMNC) algorithm and identified that UBE2O, ubiquitin-like modifier-activating enzyme 6 (UBA6), apoptosis-resistant e3 ubiqutin protein ligase 1 (AREL1), mex-3 RNA binding family member C (MEX3C), collagen type IV alpha 2 (COL4A2), laminin subunit alpha 4 (LAMA4), placental growth factor (PGF), beta-transduction repeat containing E3 ubiquitin protein ligase (BTRC), ankyrin repeat and SOCS box containing 4 (ASB4), collagen type V alpha (COL5A) are the ten hub genes among the upregulated genes (Fig. 4f). The KEGG pathway of the ten hub genes was analyzed and constructed using the online tool NetworkAnalyst (https://www.networkanalyst.ca/). The KEGG pathways of the ten hub genes were Ubiquitin mediated proteolysis, Focal adhesion, extracellular matrix (ECM)-receptor interaction, PI3K-AKT signaling pathway, Small cell lung cancer, Amoebiasis, Pathway in cancer, Circadian rhythm, African trypanosomiasis, Hedgehog signaling pathway, and Shigellosis (Additional file 1: Fig. S1B). In addition, quantitative reverse transcription PCR (qRT-PCR) demonstrated that the levels of UBE2O mRNA in saliva-Exos samples were significantly higher than in the saliva samples (Fig. 4g). HuvECs treated with saliva-Exos exhibited a higher level of UBE2O than cells treated with PBS or saliva (Fig. 4h, i). Collectively, these data suggest that UBE2O is one of the hub genes in saliva-Exos upregulated genes.

### UBE2O mediates the positive effects of saliva-Exos on HUVECs

**Fig. 5:**
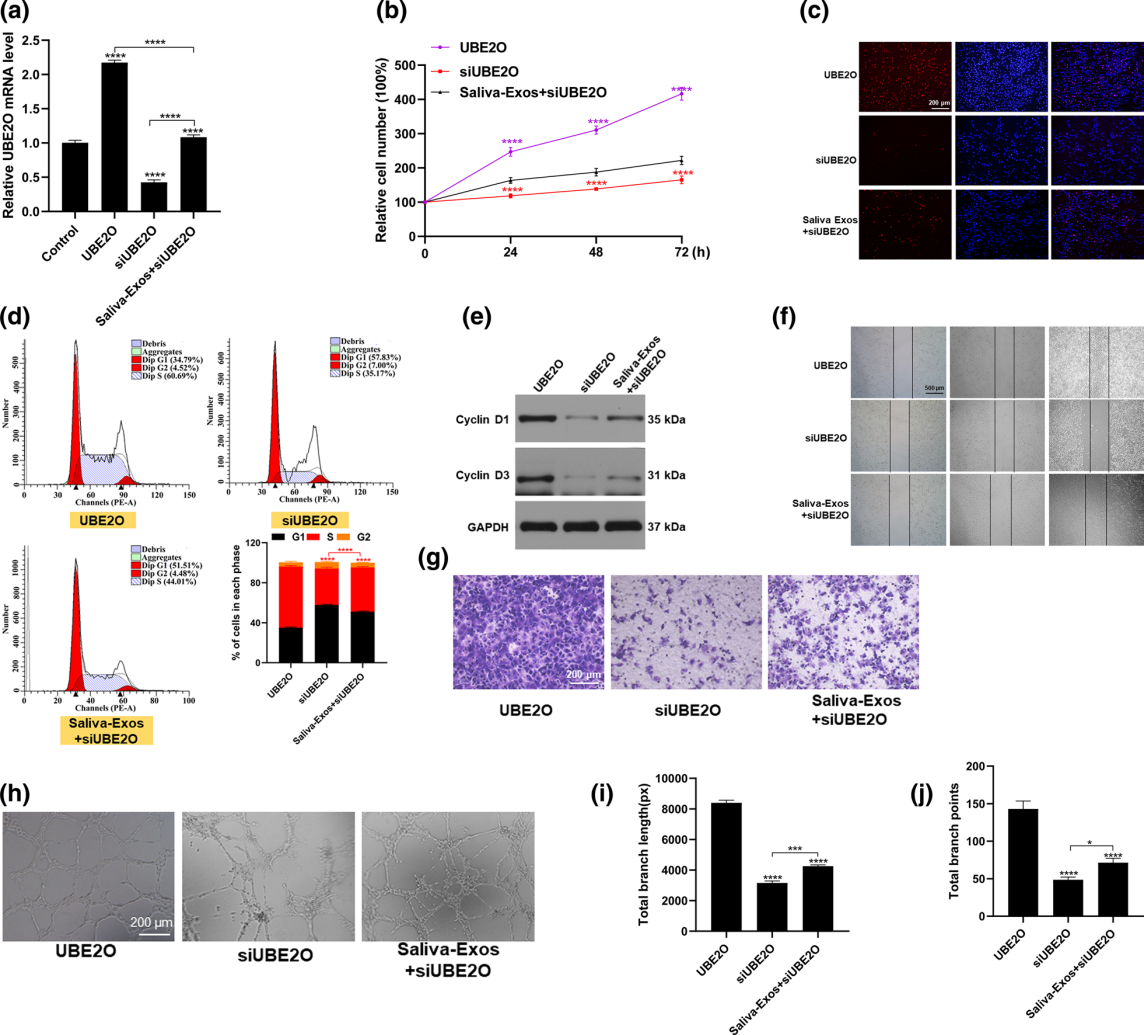
UBE2O promotes HUVEC function in vitro. **a** The UBE2O mRNA level was assessed by qRT-PCR after HUVECs were incubated with indicated treatments. **b**, **c** Proliferation of HUVECs after treatment with PBS, saliva, and saliva-Exos, measured by the CCK8 and EdU assays. **d** Distribution of HUVECs in the cell cycle assessed by flow cytometry. **e**–**g** Expression of cell cycle-related proteins determined by Western blotting. Evaluation of the migration of HUVECs by wound scratch assay and transwell assay. **h** Representative images of the tube formation assay in HUVECs treated with PBS, UBE2O, siUBE2O, and the combination of saliva-Exos and siUBE2O. **i**, **j** Quantitative analysis of total branch length and total number of branching points. *p < 0.05, **p < 0.01, ***p < 0.001

The effect of UBE2O on HUVECs in vitro was explored. Firstly, the UBE2O mRNA level was significantly higher in the UBE2O group, while its level was decreased in the small interfering RNA UBE2O (siUBE2O) group (Fig. 5a). Then, our data showed that the exposure of HUVECs to UBE2O enhanced their proliferation (Fig. 5b, c), and increased expression of cell cycle-related proteins, promoting cell entry to the S stage and (Fig. 5d, e). In agreement with these results, HUVEC migration was accelerated after treatment with UBE2O (Fig. 5f, g). UBE2O overexpression also promoted tube formation by HUVECs (Fig. 5h–j). No surprisingly, treatment of cells with siUBE2O produced opposite effects on the function of HUVECs, as documented by decreased proliferation, migration, and tube formation. Moreover, the beneficial effect of saliva-Exos was ablated by knock down of UBE2O in HUVECs. Together, these findings indicate that UBE2O is the primary mediator of the beneficial effects of saliva-Exos on HUVEC function.

### SMAD6/BMP2 axis is key to UBE2O-mediated HUVEC function

**Fig. 6:**
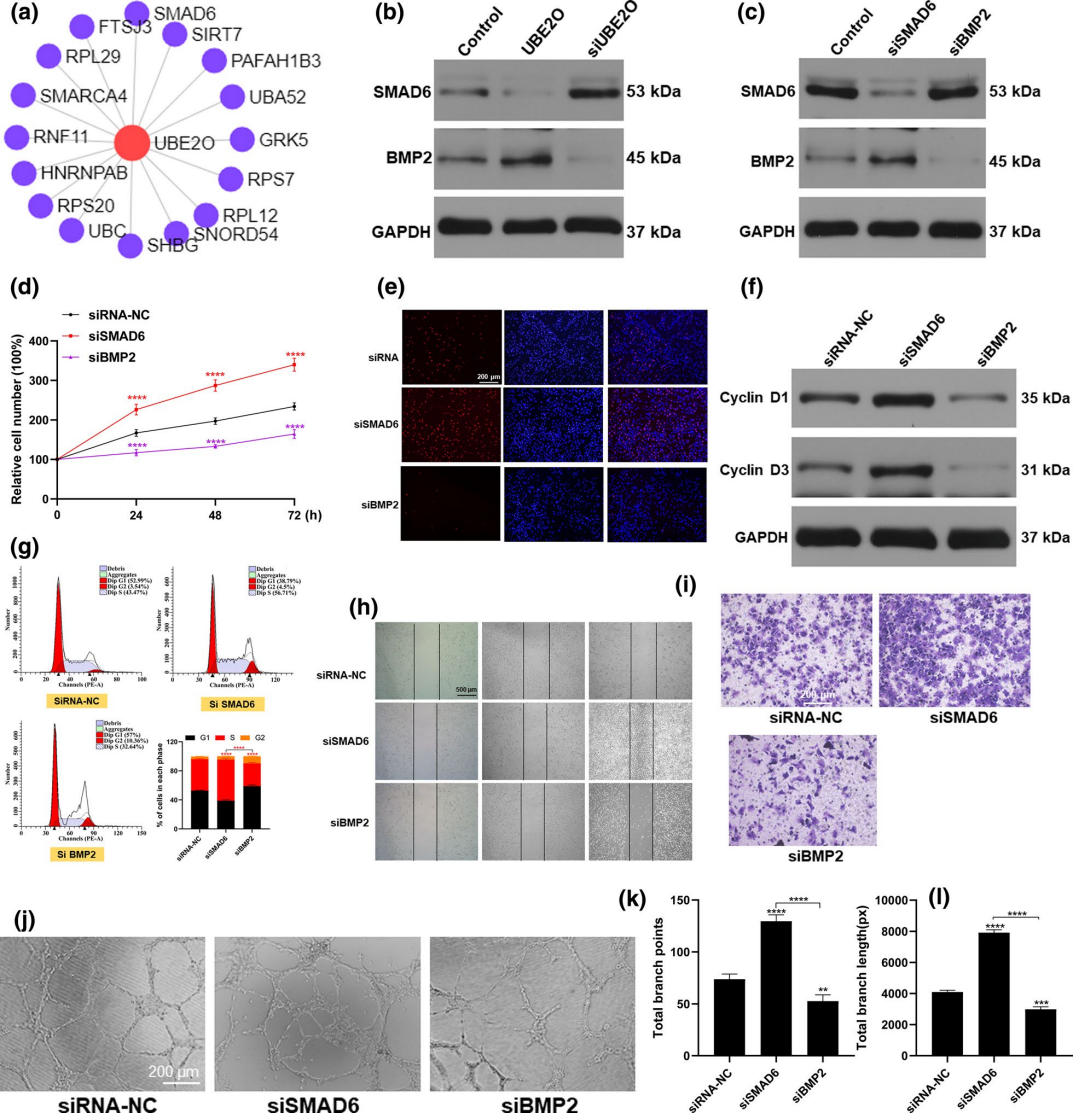
UBE2O mediates HUVEC function by suppressing the SMAD6/BMP2 pathway. **a** Network map identifying UBE2O target genes. **b** Assessment of the expression of SMAD6 and BMP2 by Western blotting. **c** Expression of SMAD6 and BMP2 in HUVECs after indicated treatments evaluated by Western blotting. **d**, **e** Proliferation of HUVECs evaluated by the CCK8 and EdU assays. **f** Detection of the cell cycle-related proteins cyclin D1 and cyclin D3, by Western blotting. **g** Flow cytometry analysis of the cell cycle. **h**, **i** HUVEC migration evaluated by wound scratch and transwell assay. **j** Representative images of the tube formation by HUVECs after indicated treatments. **k**, **l** Quantitative analysis of total branch length and total number of branching points. *p < 0.05, **p < 0.01, ***p < 0.001

To explore the gene regulatory networks of UBE2O in skin tissue, we constructed skin-type specific networks and identified that sixteen genes are regulated by UBE2O, resulting in 16 putative genes (Fig. 6a). Previous studies reported that UBE2O can target SMAD6 for ubiquitination and degradation [13]. Therefore, to determine whether UBE2O suppresses the expression of SMAD6 in HUVECs, the level of SMAD family member 6 (SMAD6) was assessed by Western blotting. As shown in Fig. 6b, the expression level of SMAD6 in HUVECs was significantly decreased after UBE2O treatment, while its level was increased after silencing UBE2O. Moreover, SMAD6 knockdown decreased the level of SMAD6 in HUVECs (Fig. 6c). SMAD6 silence significantly enhanced HUVEC function, as evidenced by the increased proliferation and migration of HUVECs in the small interfering RNA SMAD6 (siSMAD6) group (Fig. 6d–i). SMAD6 silence also induced tube formation by HUVECs (Fig. 6j–l).

SMAD/BMP pathway has been documented to be one of the main regulators of angiogenesis [14]. To identify the underlying mechanism of SMAD6-mediated angiogenesis, changes in the expression of BMP2 in HUVECs after SMAD6 knockdown were evaluated. As shown in Fig. 6c, suppressing SMAD6 resulted in BMP2 upregulation. Moreover, knockdown of bone morphogenetic protein 2 (BMP2) significantly inhibited the functions of HUVECs (Fig. 6d–l). Collectively, these data suggest that SMAD6/BMP2 pathway is involved in UBE2O-mediated HUVEC angiogenesis.

### UBE2O promotes wound healing in vivo

**Fig. 7:**
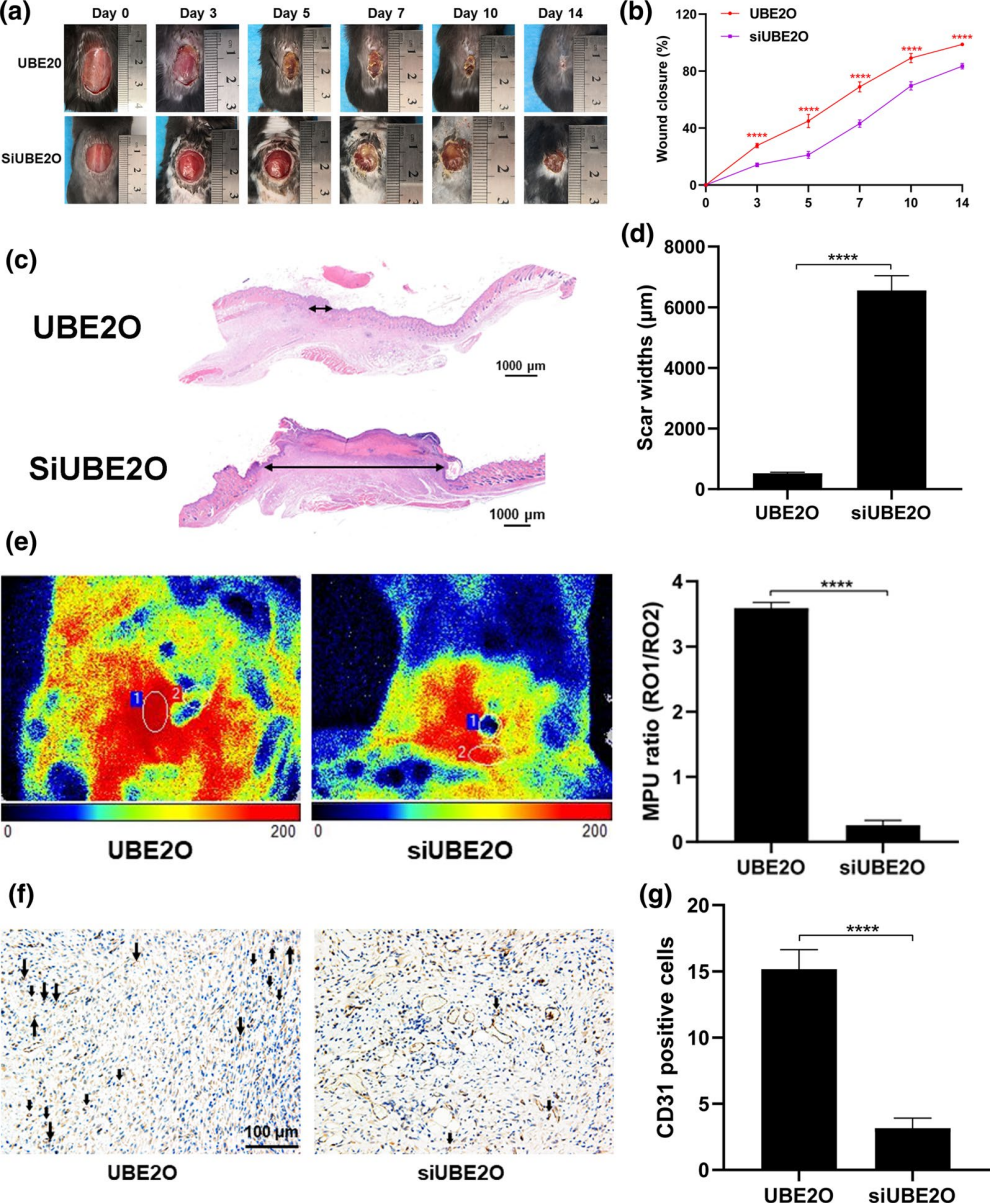
UBE2O promotes wound healing in vivo. **a** Representative images of wounds among two groups at indicated days. **b** A higher wound healing rate was seen in the UBE2O group than in the siUBE2O group. **c** H&E images of the wound sections in the two groups. **d** Smaller scar widths were seen in the UBE2O than in the siUBE2O group. **e** Blood flow at the wound sites assessed by laser speckle contrast imaging and quantification of the MPU ratio in the two groups. **f** Representative images of CD31-stained wound sections. **g** Quantification of the number of CD31-positive cells in the two groups. ****p < 0.0001; n = 6 per group

To investigate the effects of UBE2O on wound healing, UBE2O or siUBE2O was injected around the wound site. The rate of wound healing was faster in the UBE2O group than in the siUBE2O group (Fig. 7a, b). H&E staining documented that the scar width was the smallest in the UBE2O group (Fig. 7c, d). Laser speckle contrast imaging showed that blood flow was significantly higher in the UBE2O group than in the siUBE2O group, as supported by a higher MPU ratio in the UBE2O group (Fig. 7e). Additionally, the number of CD31-positive cells in the wound of UBE2O-treated mice was higher than in the siUBE2O-treated mice (Fig. 7f, g). Collectively, these findings indicate that UBE2O promotes wound healing in vivo.

## Discussion

Interest in the application of saliva-Exos as potential diagnostic and prognostic biomarkers of cancer is increasing [15]. Few studies have, however, focused on the therapeutic effects of saliva-Exos. The present study demonstrated that treatment of C57/BL6 mice with saliva-Exos promotes wound healing. Although a similar effect is is seen with use of saliva, efficacy is significantly higher with saliva-Exos. An underdeveloped vascular network has been widely regarded as the main contributor to delayed wound healing, and promotion of angiogenesis appears to be an important strategy to promote wound repair [16]. The role of Exos as a catalyst of angiogenesis has recently became gained acceptance. Plasma- or cell-derived Exos have the potential to induce formation of new vessels, hence promoting wound healing [17, 18]. The current in vivo study documented that saliva-Exos can also achieve these effects. Moreover, the in vitro experiments demonstrated that saliva-Exos promote HUVEC proliferation, migration, and tube formation. Therefore, use of saliva-Exos as a potential biological therapy for wound healing appears promising.

Several methods to isolate exosomes from blood, cells, and saliva have been reported [19]. The current data showed that ultracentrifugation-based isolation is adequate for the isolation of exosomes from saliva, as evidenced by the presence of particles of 30–150 nm in diameter and expression of markers of exosomal membranes, CD81 and TSG101. It is well-documented that Exos can be taken up by proximal or distant cells, modifying the function of target cells by transporting small molecules [20, 21]. The present study demonstrated that saliva-Exos can be taken up by HUVECs.

With the advancement of technology, bioinformatic analysis has flourished. RNA-sequencing (RNA-Seq) is one of the techniques that enables researchers to better understand the transcriptome of a vast range of organisms [22]. In the present study, we obtained RNA-Seq data of saliva-Exos from the GEO database and found that there were 312 upregulated DEGs in saliva-Exos samples when compared to the saliva sample. The large number of DEGs can be transferred into target cells along with Exos, thereby driving the diverse function of cells. In the present study, both saliva and saliva-Exos enhanced HUVEC function, with saliva-Exos having the greatest effect. We support that the synergistic effect of those genes is the main contributor to saliva-Exos-induced HUVEC activation. Among them, hub genes have been considered functionally significant [23]. Using the DMNC algorithm, we identified ten hub genes and their KEGG pathway was further analyzed. The result suggests that the Ubiquitin mediated proteolysis is the main underlying pathway, leading us to consider the importance of UBE2O, UBA6 and BTRC on saliva-Exos-mediated HUVECs activation.

The ubiquitin-proteasome system has a critical function in regulation of cellular function. UBE2O is an E2/E3 hybrid ubiquitin-protein ligase, which participates in ubiquitin mediated proteolysis [24]. In the current study, we first demonstrated that UBE2O levels are significantly higher in the saliva-Exos samples than the saliva samples. In vitro and in vivo studies showed that overexpression of UBE2O promotes HUVEC function and wound healing, while knockdown of UBE2O impairs function. Previous studies reported that UBE2O ubiquitinates AMPKα2 in skeletal muscle cells and tumor cells [25, 26]. Using the tissue-specific PPI network analysis, sixteen genes were identified as targets of UBE2O on skin tissue. Previous studies reported that UBE2O can target SMAD6 during bone morphogenetic protein signaling, [13] but its role in wound healing has yet to be elucidated. In the present study, we demonstrated that UBE2O decreases SMAD6 level in HUVECs. SMADs represent a family of proteins that can be divided into three classes: R-SMADs (receptor-activated SMADs), Co-SMADs (common SMADs) and I-SMADs (inhibitory SMADs). SMADs are one of the main regulators of physiological and pathological blood vessel formation. Overexpression of R-SMADs, including SMAD1/5/8, in endothelial cells enhances angiogenesis [27, 28]. However, I-SMADs, such as SMAD6, inhibit R-SMAD phosphorylation and nuclear translocation, suppressing angiogenesis [29, 30]. In agreement with these previous findings, data obtained in the present study indicate that knockdown of SMAD6 significantly enhanced HUVECs function. Knock down of SMAD6 could increase BMP2 expression level in HUVECs. It has been reported that BMP2 is one of the main pro-angiogenic cytokines [31], and knockdown of BMP2 in the present study suppressed the proliferation of HUVECs and impaired angiogenesis. Together, the results of the present and previous studies unequivocally demonstrate the role of the UBE2O-SMAD6-BMP2 axis in the regulation of angiogenesis.

The unique characteristics of Exos, such as natural transportation properties and good biocompatibility, have recently attracted a significant amount of attention [32]. Exosomes can transport their cargo, such as protein, lipids, and nucleic acids, into the target cells, thereby regulating biological processes [33]. Extensive efforts have been made to load exosomes with nanomaterials in order to deliver them to target organs and improve organ function [34]. Engineered exosomes loaded with specific proteins have also been demonstrated to act as effective therapeutic agents for a variety of diseases [35]. Sharma and coworkers [36] reported that saliva-Exos have unique features, such as distinct elastic properties and substructures carrying specific transmembrane receptors. These unique features along with wide availability of saliva-Exos make them an excellent agent for tissue engineering.

Wound healing is a complex process that involves various cells and cytokines. The present study focused mainly on the role of saliva-Exos on HUVECs, but future studies should address the impact of saliva-Exos on other cell types, such as keratinocytes and fibroblasts. In addition, saliva-Exos contain multiple types of molecules that may play catalytic roles in wound healing. Specifically, exploring the effect of saliva-Exos and UBE2O on the healing of diabetic and chronic wounds may be promising.

## Conclusions

**Fig. 8:**
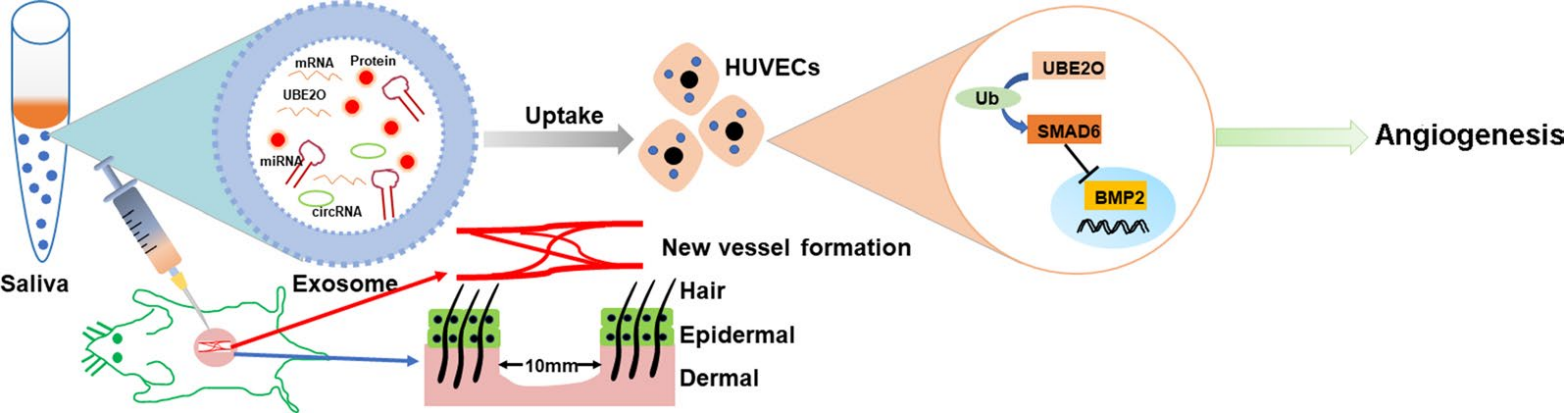
UBE2O mRNA is the one of the main cargo of Saliva-Exos, which could be transport into HUVECs, thereby decreases the level of SMAD6, subsequently activating BMP2, which, in turn, induces angiogenesis in vitro and promotes wound repair in vivo

In summary, the current work highlighted that saliva-Exos enhance HUVEC function through UBE2O delivery. Overexpression of UBE2O decreases the SMAD6 level, leading to upregulation of BMP2 expression and, consequently, promotion of angiogenesis in vitro and acceleration of wound healing in vivo (Fig. 8). These findings indicate that saliva-Exos are a potential promising agent for wound therapy. Furthermore, since upregulation of UBE2O accelerates angiogenesis, use of nanomaterials combined with UBE2O may enhance wound healing.

## Methods

### Cell culture

HUVECs were purchased from the Cell Bank of the Chinese Academy of Science (Shanghai, China). The cells were cultured in RPMI1640 supplemented with 10% FBS, at 37 °C, in the presence of 5% CO_2_. The lentiviral UBE2O was constructed by GenePharma (Shanghai, China). Briefly, the cDNA fragment of UBE2O was generated with the following primers: 5′-TCGAGCTCAAGCTTATGACCTCAGCCGACGT GATG-3′ (forward) and 5′-CTCACCATGACCCATGACCGGTGGATCCTCCT TGTCCTCTGTGCACTCCG-3′ (reverse) and inserted into pLVX-AcGFP lentiviral vector within the EcoRI and BamHI sites. The siRNA UBE2O and siRNA SMAD6was purchased from Santa Cruz Biotechnology, Inc, respectively (Dallas, Texas, USA, #sc-94199, #sc-38380). Cells were transfected with the RNAs using lipofectamine 3000 (Invitrogen, USA). UBE2O was transfected at a concentration of 1 μg/ml, and the siRNA UBE2O was transfected at a concentration of 50 nM. Besides, cells were treated with 100 μg/ml Saliva-Exos and 20% saliva. All the cell experiments were repeated in triplicates. The in vitro and in vivo properties of saliva and saliva-Exos were verified by three independent experiments through randomly using three author-derived saliva and saliva-Exos.

### Saliva collection

Unstimulated Saliva was collected from the six authors (BM, LC, YX, CY, HX, JL). All authors were healthy and the average age of them was 28.33 ± 2.58. The authors wash their mouth thoroughly with water before the collection of saliva in the morning between 8:00 and 9:00 am. Saliva samples were centrifuged at 2600 g for 5 min at 4 °C. Then, saliva supernatant removed to a new sterile tube and was further filter sterilized through a 0.22 μm filter to remove pathogens. Then the saliva was diluted in PBS to concentration of 40% saliva, which further diluted in the culture medium (1:1) to final concentrations of 20% saliva.

### Isolation and identification of saliva-Exos

Cell-free saliva samples (10 ml) were collected from the authors. The specimens were centrifuged at 2,000 g for 30 min, and the resulting supernatant was centrifuged for 45 min at 12,000 g at 4°C. The obtained supernatant was filtered through a 0.45 μm filter membrane, followed by centrifuging at 110,000 g for 70 min. The supernatant was discarded, and the pellets were resuspended in PBS, and re-centrifuged at 110,000 g for 70 min. Finally, the particles were resuspended with cold PBS for further analysis. The morphology of particles was evaluated using transmission electron microscopy (TEM) (Tecnai Spirit T12, FEI) and the sizes were analyzed by NanoSight (Flow NanoAnalyzer, NanoFCM, Xiamen, China). Briefly, 10 μl saliva-Exos was loaded on carbon-coated copper grids for 1 min, then 10 μl phosphotungstic acid was added to the copper grids for another 1 min. Grids were viewed using TEM and photographed for further analysis.

### Bioinformatics analysis and Protein-protein interaction (PPI) network construction

The RNA-sequencing data (GSE50700) were obtained from the Gene Expression Omnibus (GEO) database. The data included three samples of saliva and three samples of saliva-Exos. The online tool GEO2R was used to identify differentially expressed genes (DEGs). The threshold of DEGs was set as | log2 fold-change (FC)| ≥ 3.5 with p-value < 0.05. The protein-protein interaction (PPI) network of the upregulated genes with a putative target of skin tissue were constructed utilizing the online tool NetworkAnalyst (https://www.networkanalyst.ca/). Furthermore, the PPI network between UBE2O and the putative target in skin tissue were also constructed by NetworkAnalyst.

### Gene Ontology (GO) enrichment and Kyoto Encyclopedia of Genes and Genomes (KEGG) pathway analysis

To understand the biological function of upregulated DEGs, the Annotation, Visualization, and Integrated Discovery (DAVID, http://david.abcc.ncifcrf.gov/) resource was used to analyze their GO and KEGG pathway. The top ten pathways derived from functional enrichment of each GO subset and KEGG pathway were illustrated in a bubble diagram with the ggplot2 tool of R package.

### Hub gene selection and KEGG pathway construction

The novel Cytoscape plugin, cytoHubba, was used to rank the genes contained in the network of the upregulated DEGs. The Density of Maximum Neighborhood Component (DMNC) algorithm was selected from the 11 topological analysis methods provided by CytoHubba to identify the top ten hub genes. Furthermore, the KEGG pathway of the ten hub genes were analyzed and constructed using the online tool NetworkAnalyst (https://www.networkanalyst.ca/).

### Cell counting kit 8 (CCK8) assay

HUVECs were seeded into 96-well culture plates at a density of 5 × 10^3^ cells per well and treated with PBS (control), saliva or saliva-Exos (100 μg/ml). After incubation at 37 °C for 24, 48, and 72 h, 10 μl of CCK8 reagent was added to each well. Two hours later, the absorbance of each well was measured using a microplate reader at 450 nm.

### EdU assay

HUVECs were seeded into 24-well culture plates at a density of 2 × 10^5^ cells/well and treated with PBS, saliva, or saliva-Exos (100 μg/ml) for 24 h. Subsequently, EdU staining was performed according to the manufacturer’s protocol (Sigma-Aldrich, St. Louis, MO, USA).

### Scratch wound healing assay

After treatment with the indicated reagents, a monolayer of HUVECs grown in six-well plates was scratched with a sterile 10 μl pipette tip to form wounds in the sheet of cells. The plates were then incubated for 12 and 24 h, and the cell-free wound area was photographed under an inverted microscope.

### Transwell migration assay

The 24-well culture plates containing 8 μm pore-sized filters were used to assess cell migration. 1 × 10^4^ cells were seeded into the upper chamber. 500 μl medium containing Exos or other reagents were added into the lower chamber. After incubation for 12 h, the number of migrated cells was observed under an optical microscope.

### Cell cycle analysis

Cell cycle progression was evaluated by propidium iodide (PI) staining using a flow cytometer according to the manufacturer’s protocol (#KGA511-KGA512, KeyGEN Biotech, Jiangsu, China).

### Tube formation assay

Each well of a 96-well plate was filled with 50 μl of Growth Factor Reduced Matrigel (BD Biosciences, NJ, USA). After incubation for 30 min, 2 × 10^4^ HUVECs were seeded into the wells, and tube formation was observed 12 h later under an inverted microscope.

### Western blotting

Total protein was extracted from saliva, saliva-Exos and cells. Then 40 μg proteins of each sample was separated by 10% SDS-PAGE, and transferred onto PVDF membranes. The membranes were incubated with primary antibodies at 4 °C for overnight, and with horseradish-peroxidase-conjugated secondary antibodies at 37 °C for 1 h. The following antibodies were used: anti-CD81 (1:1000, Abcam, MA, USA, #ab109201), anti-TSG101 (1:1000, Abcam, MA, USA, #ab125011), anti-Calnexin, (1:1000, Abcam, MA, USA, #ab125011), anti-Cyclin D1 (1:1000, Abcam, MA, USA, #ab40754), anti-Cyclin D3 (1:1000, CST, USA, #2936), anti-UBE2O (1:500, Abcam, MA, USA, #ab254592), anti-SMAD6 (1:1000, Abcam, MA, USA, #ab80049), anti-BMP2 (1:1000, Abcam, MA, USA, #ab14933) and anti-GADPH (1:10,000, Abcam, USA, #ab37168).

### Quantitative real-time PCR (qRT-PCR)

Total RNA was collected using TRIzol Reagent (Invitrogen), and 1 μg of total RNA was transcribed into cDNA. qRT-PCR was performed using the StepOne™ Real-Time PCR (Life Technologies, Carlsbad, CA, USA). Relative gene expression was calculated using the 2^−ΔΔCt^ method, and GAPDH was used to normalize mRNA levels. Primer sequences used for qRT-PCR were as follows: UBE2O, Forward: 5′-ACATCCGCTCCAACGAC-3′, Reverse: 5′-GCTGGTGCTGCCTTCTAC-3′, BMP2, Forward: 5’-ATGGATTCGTGGTGGAAGTG-3’, Reverse: 5’-GTGGAGTTCAGATG ATCAGC-3’; SMAD6: Forward, 5’-ACGGTGACCTGCTGTCTCTT-3’, Reverse: 5’-AGCGAGTACGTGACCGTCTT-3’, GAPDH, 5′-CCAGCCGAGCCACATCGCTC-3′ and 5′-ATGAGCCCCAGCCTTCTCCAT-3′.

### Mouse skin wound model

C57BL/6 male mice (6–8 weeks old) were purchased from the Center of Experimental Animals, Tongji Medical College, Huazhong University of Science and Technology. The mice were anesthetized with pentobarbital sodium (50 mg/Kg), and one full-thickness excisional skin wound (10 mm in diameter) was created on the dorsum of each mouse. Subsequently, the mice were randomly divided into five groups: mice treated with PBS (100 μl), saliva (100 μl), saliva-Exos (100 μg saliva-Exos in 100 μl PBS), plasmid UBE2O UBE2O (in PBS), or siUBE2O (in PBS). The concentration of UBE2O and siUBE2O in animal experiments was 100 μl of a 20 μmol/l UBE2O or siUBE2O in PBS. The solutions were subcutaneously injected in four sites adjacent to the wound (25 μl/site). The wounds were digitally photographed at days 0, 3, 7, 10, and 14 post-wounding. The mice were sacrificed at day 14, and skin samples were harvested for further analysis. The area of the wound was measured using ImageJ^®^ software (version 1.52a; Media Cybernetics, Bethesda, MA, USA). Wound healing was calculated using the following formula:

$$Wound \;healing = \frac{Wound \; area \; on \; Day \; n}{Wound \; area \; on \; Day \;0} \times 100$$

where n is day 0, 3, 7, 10, and 14. All protocols involving animals were reviewed and approved by the Animal Care and Use Committee of the Tongji Medical College, Huazhong University of Science and Technology.

### Assessment of blood flow in the wound site

Ten days after the operation, laser speckle contrast imaging (LSCI) (PERIMED Ltd, Stockholm, Sweden) was used to evaluate blood flow in the wound. The mean perfusion units (MPU) ratio was calculated by comparing the MPU per mm^2^ in the wound area (ROI-1) with the MPU per mm^2^ in an area adjacent to the wound (ROI-2).

### Hematoxylin and eosin (H&E) staining and immunohistochemistry

Tissue samples from Day 14 containing the wound region were collected, fixed in 4% buffered paraformaldehyde and embedded in paraffin. 4 μm thick sections were prepared and stained with H&E. CD31 was detected using immunofluorescence staining; briefly, sections were blocked in 1% BSA for 30 min and incubated overnight with anti-CD31 (1:50, Abcam, #ab28364). Subsequently, the sections were incubated with secondary antibody for 1 h. CD31-positive cells were quantified from at least three randomly selected high power fields per section. All slides were independently evaluated by three observers blinded to the treatment.

### Statistical analysis

Data are shown as mean ± standard deviation (SD). All analyses were performed using GraphPad Prism version 8.00 for MacOS (GraphPad Software, La Jolla California USA). Student’s *t*-test was used to compare the differences between two groups, and one-way analysis of variance (ANOVA) with Tukey’s *post hoc* test was used to analyze the differences in more than two groups. P < 0.05 was considered to indicate statistically significant difference.

## Supplementary information

**Additional file 1: Fig. S1.** (A) 3D network viewer displaying a force-directed PPI network between skin tissue with upregulated genes of saliva-Exos. (B) The KEGG pathway of each of ten hub genes was analyzed and constructed by the online tool NetworkAnalyst.

## Data Availability

All data generated or analyzed during this study are included in this published article.

## References

[1] Martinengo L, Olsson M, Bajpai R, Soljak M, Upton Z, Schmidtchen A, Car J, Jarbrink K (2019). Prevalence of chronic wounds in the general population: systematic review and meta-analysis of observational studies. Ann Epidemiol.

[2] Sen CK (2019). Human wounds and its burden: an updated compendium of estimates. Adv Wound Care.

[3] Zhu Z, Liu Y, Xue Y, Cheng X, Zhao W, Wang J, He R, Wan Q, Pei X (2019). Tazarotene released from aligned electrospun membrane facilitates cutaneous wound healing by promoting angiogenesis. ACS Appl Mater Interfaces.

[4] Su CH, Li WP, Tsao LC, Wang LC, Hsu YP, Wang WJ, Liao MC, Lee CL, Yeh CS (2019). Enhancing microcirculation on multitriggering manner facilitates angiogenesis and collagen deposition on wound healing by photoreleased no from hemin-derivatized colloids. ACS Nano.

[5] Wang P, Huang S, Hu Z, Yang W, Lan Y, Zhu J, Hancharou A, Guo R, Tang B (2019). In situ formed anti-inflammatory hydrogel loading plasmid DNA encoding VEGF for burn wound healing. Acta Biomater.

[6] Playford RJ, Macdonald CE (1997). Growth factors in saliva. Lancet.

[7] Torres P, Diaz J, Arce M, Silva P, Mendoza P, Lois P, Molina-Berrios A, Owen GI, Palma V, Torres VA (2017). The salivary peptide histatin-1 promotes endothelial cell adhesion, migration, and angiogenesis. FASEB J..

[8] Xiao H, Wong DT (2012). Proteomic analysis of microvesicles in human saliva by gel electrophoresis with liquid chromatography-mass spectrometry. Anal Chim Acta.

[9] Niu Q, Bao C, Cao X, Liu C, Wang H, Lu W (2019). Ni-Fe PBA hollow nanocubes as efficient electrode materials for highly sensitive detection of guanine and hydrogen peroxide in human whole saliva. Biosens Bioelectron.

[10] Liu C, Zhang W, Li Y, Chang J, Tian F, Zhao F, Ma Y, Sun J (2019). Microfluidic sonication to assemble exosome membrane-coated nanoparticles for immune evasion-mediated targeting. Nano Lett.

[11] Luo ZW, Li FX, Liu YW, Rao SS, Yin H, Huang J, Chen CY, Hu Y, Zhang Y, Tan YJ, Yuan LQ, Chen TH, Liu HM, Cao J, Liu ZZ, Wang ZX, Xie H (2019). Aptamer-functionalized exosomes from bone marrow stromal cells target bone to promote bone regeneration. Nanoscale..

[12] Che Y, Shi X, Shi Y, Jiang X, Ai Q, Shi Y, Gong F, Jiang W (2019). Exosomes derived from miR-143-overexpressing MSCs inhibit cell migration and invasion in human prostate cancer by downregulating TFF3. Mol Ther Nucleic Acids..

[13] Zhang X, Zhang J, Bauer A, Zhang L, Selinger DW, Lu CX, Ten Dijke P (2013). Fine-tuning BMP7 signalling in adipogenesis by UBE2O/E2-230 K-mediated monoubiquitination of SMAD6. EMBO J.

[14] Larrivee B, Prahst C, Gordon E, del Toro R, Mathivet T, Duarte A, Simons M, Eichmann A (2012). ALK1 signaling inhibits angiogenesis by cooperating with the Notch pathway. Dev Cell.

[15] Chiabotto G, Gai C, Deregibus MC, Camussi G (2019). Salivary extracellular vesicle-associated exRNA as cancer biomarker. Cancers (Basel).

[16] Okonkwo UA, Di Pietro LA (2017). Diabetes and wound angiogenesis. Int J Mol Sci.

[17] Chen CY, Rao SS, Ren L, Hu XK, Tan YJ, Hu Y, Luo J, Liu YW, Yin H, Huang J, Cao J, Wang ZX, Liu ZZ, Liu HM, Tang SY, Xu R, Xie H (2018). Exosomal DMBT1 from human urine-derived stem cells facilitates diabetic wound repair by promoting angiogenesis. Theranostics..

[18] Hu Y, Rao SS, Wang ZX, Cao J, Tan YJ, Luo J, Li HM, Zhang WS, Chen CY, Xie H (2018). Exosomes from human umbilical cord blood accelerate cutaneous wound healing through miR-21-3p-mediated promotion of angiogenesis and fibroblast function. Theranostics..

[19] Li P, Kaslan M, Lee SH, Yao J, Gao Z (2017). Progress in exosome isolation techniques. Theranostics..

[20] Chen G, Huang AC, Zhang W, Zhang G, Wu M, Xu W, Yu Z, Yang J, Wang B, Sun H, Xia H, Man Q, Zhong W, Antelo LF, Wu B, Xiong X, Liu X, Guan L, Li T, Liu S, Yang R, Lu Y, Dong L, McGettigan S, Somasundaram R, Radhakrishnan R, Mills G, Lu Y, Kim J, Chen YH, Dong H, Zhao Y, Karakousis GC, Mitchell TC, Schuchter LM, Herlyn M, Wherry EJ, Xu X, Guo W (2018). Exosomal PD-L1 contributes to immunosuppression and is associated with anti-PD-1 response. Nature.

[21] Holmes D (2016). Adipose tissue: adipocyte exosomes drive melanoma progression. Nat Rev Endocrinol..

[22] Corley SM, MacKenzie KL, Beverdam A, Roddam LF, Wilkins MR (2017). Differentially expressed genes from RNA-Seq and functional enrichment results are affected by the choice of single-end versus paired-end reads and stranded versus non-stranded protocols. BMC Genomics..

[23] Seo CH, Kim JR, Kim MS, Cho KH (2009). Hub genes with positive feedbacks function as master switches in developmental gene regulatory networks. Bioinformatics.

[24] Chen S, Yang J, Zhang Y, Duan C, Liu Q, Huang Z, Xu Y, Zhou L, Xu G (2018). Ubiquitin-conjugating enzyme UBE2O regulates cellular clock function by promoting the degradation of the transcription factor BMAL1. J Biol Chem.

[25] Vila IK, Park MK, Setijono SR, Yao Y, Kim H, Badin PM, Choi S, Narkar V, Choi SW, Chung J, Moro C, Song SJ, Song MS (2019). A muscle-specific UBE2O/AMPKalpha2 axis promotes insulin resistance and metabolic syndrome in obesity. JCI Insight.

[26] Vila IK, Yao Y, Kim G, Xia W, Kim H, Kim SJ, Park MK, Hwang JP, Gonzalez-Billalabeitia E, Hung MC, Song SJ, Song MS (2017). A UBE2O-AMPKalpha2 axis that promotes tumor initiation and progression offers opportunities for therapy. Cancer Cell.

[27] Moya IM, Umans L, Maas E, Pereira PN, Beets K, Francis A, Sents W, Robertson EJ, Mummery CL, Huylebroeck D, Zwijsen A (2012). Stalk cell phenotype depends on integration of Notch and Smad1/5 signaling cascades. Dev Cell.

[28] Richter A, Alexdottir MS, Magnus SH, Richter TR, Morikawa M, Zwijsen A, Valdimarsdottir G (2019). EGFL7 mediates BMP9-induced sprouting angiogenesis of endothelial cells derived from human embryonic stem cells. Stem Cell Rep.

[29] Mouillesseaux KP, Wiley DS, Saunders LM, Wylie LA, Kushner EJ, Chong DC, Citrin KM, Barber AT, Park Y, Kim JD, Samsa LA, Kim J, Liu J, Jin SW, Bautch VL (2016). Notch regulates BMP responsiveness and lateral branching in vessel networks via SMAD6. Nat Commun..

[30] Hata A, Lagna G, Massague J, Hemmati-Brivanlou A (1998). Smad6 inhibits BMP/Smad1 signaling by specifically competing with the Smad4 tumor suppressor. Genes Dev.

[31] Lee E, Ko JY, Kim J, Park JW, Lee S, Im GI (2019). Osteogenesis and angiogenesis are simultaneously enhanced in BMP2-/VEGF-transfected adipose stem cells through activation of the YAP/TAZ signaling pathway. Biomater Sci..

[32] Liu C, Su C (2019). Design strategies and application progress of therapeutic exosomes. Theranostics..

[33] Pegtel DM, Gould SJ (2019). Exosomes. Annu Rev Biochem.

[34] Zhang K, Zhao X, Chen X, Wei Y, Du W, Wang Y, Liu L, Zhao W, Han Z, Kong D, Zhao Q, Guo Z, Han Z, Liu N, Ma F, Li Z (2018). Enhanced therapeutic effects of mesenchymal stem cell-derived exosomes with an injectable hydrogel for hindlimb ischemia treatment. ACS Appl Mater Interfaces.

[35] Sterzenbach U, Putz U, Low LH, Silke J, Tan SS, Howitt J (2017). Engineered exosomes as vehicles for biologically active proteins. Mol Ther.

[36] Sharma S, Rasool HI, Palanisamy V, Mathisen C, Schmidt M, Wong DT, Gimzewski JK (2010). Structural-mechanical characterization of nanoparticle exosomes in human saliva, using correlative AFM, FESEM, and force spectroscopy. ACS Nano.

