# Supplementary figures and images for: Saliva exosomes-derived UBE2O mRNA promotes angiogenesis in cutaneous wounds by targeting SMAD6
Source: J Nanobiotechnology. 2020 May 6;18:68. doi: 10.1186/s12951-020-00624-3 (PMC7203970; doi:10.1186/s12951-020-00624-3)

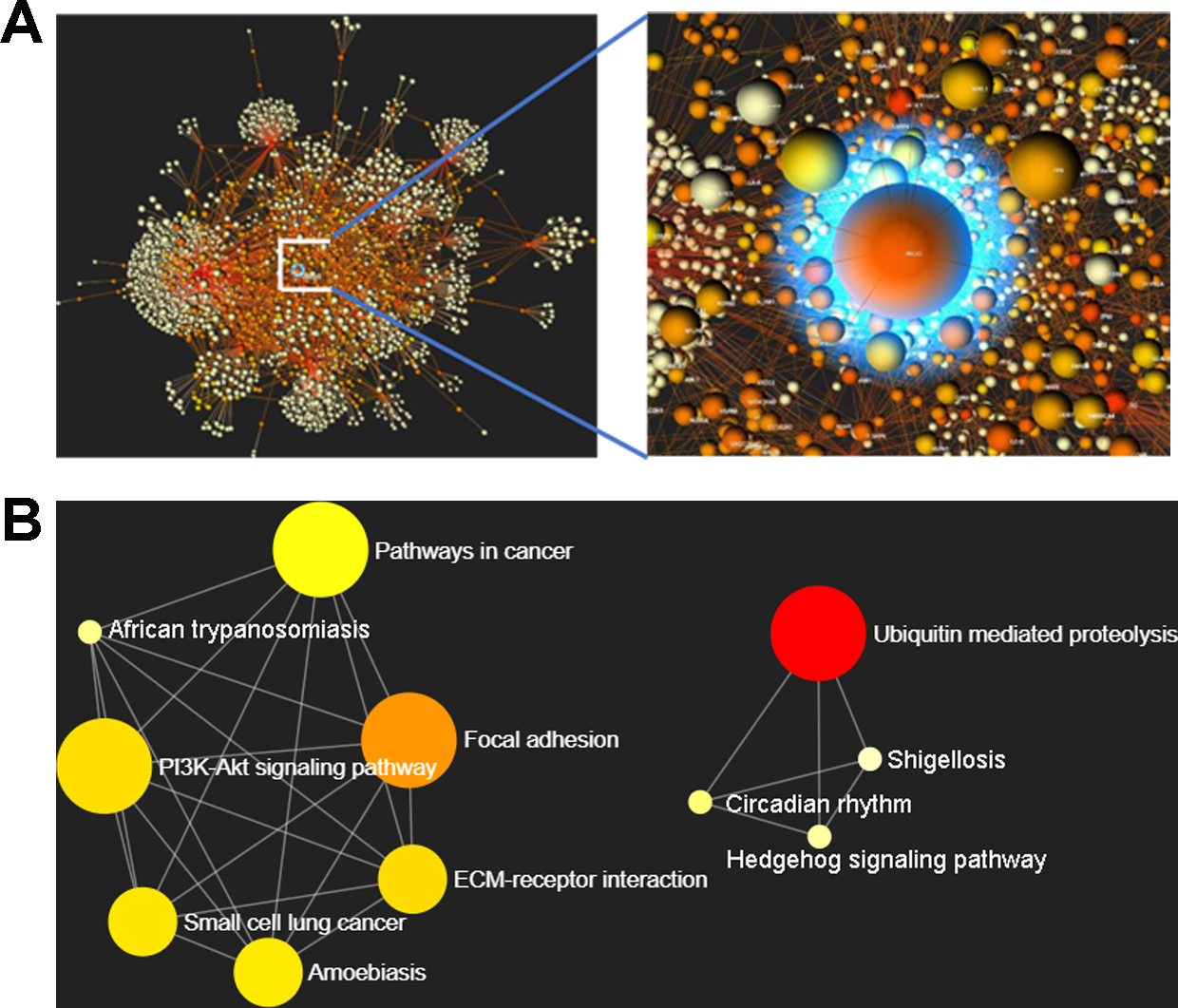

Supplement: Supplementary file 1 — Additional file 1: Fig. S1. (A) 3D network viewer displaying a force-directed PPI network between skin tissue with upregulated genes of saliva-Exos. (B) The KEGG pathway of each of ten hub genes was analyzed and constructed by the online tool NetworkAnalyst. [file 12951_2020_624_MOESM1_ESM.tif]
